# Supplementary material for: Causal Relationship between Aging and Anorexia Nervosa: A White-Matter-Microstructure-Mediated Mendelian Randomization Analysis
Source: Biomedicines. 2024 Aug 16;12(8):1874. doi: 10.3390/biomedicines12081874 (PMC11351342; doi:10.3390/biomedicines12081874)
Supplement: Supplementary file 1 [file biomedicines-12-01874-s001.zip › Supplementary Figure/Figure S4/Singe SNP plot of MR analysis for TL to ubm-b-1733.pdf]

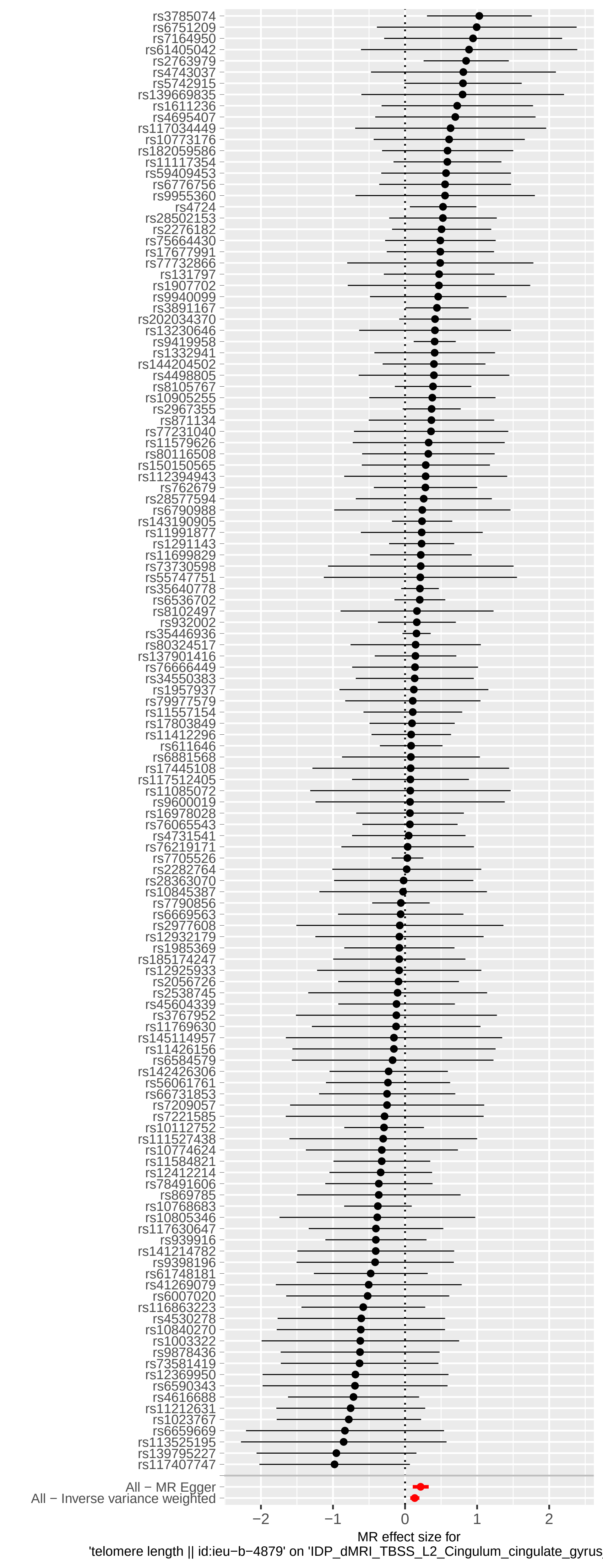
rs3785074
rs6751209
rs7164950
rs61405042
rs2763979
rs4743037
rs5742915
rs139669835
rs1611236
rs4695407
rs117034449
rs10773176
rs182059586
rs11117354
rs59409453
rs6776756
rs9955360
rs4724
rs28502153
rs2276182
rs75664430
rs17677991
rs77732866
rs131797
rs1907702
rs9940099
rs3891167
rs202034370
rs13230646
rs9419958
rs1332941
rs144204502
rs4498805
rs8105767
rs10905255
rs2967355
rs871134
rs77231040
rs11579626
rs80116508
rs150150565
rs112394943
rs762679
rs28577594
rs6790988
rs143190905
rs11991877
rs1291143
rs11699829
rs73730598
rs55747751
rs35640778
rs6536702
rs8102497
rs932002
rs35446936
rs80324517
rs137901416
rs76666449
rs34550383
rs1957937
rs79977579
rs11557154
rs17803849
rs11412296
rs611646
rs6881568
rs17445108
rs117512405
rs11085072
rs9600019
rs16978028
rs76065543
rs4731541
rs76219171
rs7705526
rs2282764
rs28363070
rs10845387
rs7790856
rs6669563
rs2977608
rs12932179
rs1985369
rs185174247
rs12925933
rs2056726
rs2538745
rs45604339
rs3767952
rs11769630
rs145114957
rs11426156
rs6584579
rs142426306
rs56061761
rs66731853
rs7209057
rs7221585
rs10112752
rs111527438
rs10774624
rs11584821
rs12412214
rs78491606
rs869785
rs10768683
rs10805346
rs117630647
rs939916
rs141214782
rs9398196
rs61748181
rs41269079
rs6007020
rs116863223
rs4530278
rs10840270
rs1003322
rs9878436
rs73581419
rs12369950
rs6590343
rs4616688
rs11212631
rs1023767
rs6659669
rs113525195
rs139795227
rs117407747
All − MR Egger
All − Inverse variance weighted
−2
−1
0
1
2
MR effect size for
'telomere length || id:ieu−b−4879' on 'IDP_dMRI_TBSS_L2_Cingulum_cingulate_gyrus_
